# A spectral library of branches for seven boreal and temperate tree species

**DOI:** 10.1016/j.dib.2025.112255

**Published:** 2025-11-08

**Authors:** Jussi Juola, Miina Rautiainen, Aarne Hovi

**Affiliations:** Department of Built Environment, School of Engineering, Aalto University, P.O. Box 14100, FI-00076 AALTO, Finland

**Keywords:** Spectroscopy, Remote sensing, Reflectance modelling, Forest

## Abstract

Branches have a distinct effect on forest canopy reflectance spectra. Spectral data on branches are therefore essential for interpretation of optical remote sensing data with radiative transfer modeling and machine learning-based approaches. This article presents laboratory-measured reflectance spectra of branches from seven boreal and temperate tree species. A total of 84 branch samples from 21 individual trees representing coniferous and broadleaf tree species were collected in southern Finland between September and November 2024. Spectral measurements were conducted under laboratory conditions using an ASD FieldSpec 4 Standard-Res spectroradiometer (351–2500 nm), combined with either a contact probe setup or an integrating sphere setup, and a halogen light source. These data support more accurate interpretations of multi- and hyperspectral remote sensing data.

Specifications Table

**Table:** 

Subject	Earth & Environmental Sciences
Specific subject area	Remote sensing; forests; radiative transfer modelling; optical spectroscopy
Type of data	Table (.csv format) Supporting materials (.pdf format)
Data collection	The data were collected in southern Finland between September and November 2024 using a spectroradiometer (ASD FieldSpec 4 Standard-Res) coupled with either a contact probe (Thorlabs Bifurcated 19-Fiber Bundle) or an integrating sphere (Avantes Avasphere-30), and a light source (Ocean Optics HL-2000). Reflectance spectra of branches from coniferous and broadleaf tree species (European aspen (*Populus tremula* L.), goat willow (*Salix caprea* L.), grey alder (*Alnus incana* (L.) Moench), silver birch (*Betula pendula* Roth), Scots pine (*Pinus sylvestris* L.), Norway spruce (*Picea abies* (L.) H. Karst), and Siberian larch (*Larix sibirica* Ledeb.)) were measured under laboratory conditions. Each measurement was accompanied by auxiliary field measurements describing the sampled branch and the tree it was detached from, e.g., tree height and branch length.
Data source location	Lohja, Finland (60° 23′N, 23°53′E) Helsinki, Finland (60° 13′N, 25° 00′E)
Data accessibility	Repository name: Mendeley Data **A dataset of branch reflectance spectra for seven boreal and temperate tree species** Data identification number: https://doi.org/10.17632/kvnx6vt8x9.2 Direct URL to data: https://data.mendeley.com/datasets/kvnx6vt8x9/2
Related research article	*none*

## 1 Value of the Data

• Existing spectral libraries for forest reflectance modeling have focused mainly on foliage, understory, and stems, leaving branches largely unrepresented.
• This is the first dataset to systematically capture the spectral characteristics of branches from seven boreal and temperate tree species under controlled laboratory conditions, representing the largest openly available and most detailed collection of branch-level hyperspectral measurements to date.
• The high-resolution spectral data can be applied in remote sensing tasks such as radiative transfer modeling and machine-learning-based interpretation of multi- and hyperspectral imagery. By improving the representation of forest spectral diversity and structural complexity, these data enhance physically-based forest reflectance models, and ecological simulations.

## 2 Background

In remote sensing, spectral libraries are collections of reference measurements describing how, e.g., different canopy elements reflect electromagnetic radiation across wavelengths. They are essential for developing and validating remote sensing methods, improving land surface models, and understanding how forests and vegetation interact with solar radiation. Because forest canopies are complex three-dimensional structures composed of leaves, stems, branches, and other elements that interact with solar radiation in diverse ways, comprehensive spectral libraries are crucial for capturing this structural and optical diversity. Among these elements, branches play a distinct role in modulating canopy reflectance, particularly in seasons or conditions where foliage is sparse or absent. Despite their importance, branches have received limited attention in spectral studies, and open spectral libraries of branches have been scarce [1,2]. To address this gap, we have produced this spectral library capturing the hyperspectral reflectance of branches from seven boreal and temperate tree species. This dataset represents the largest and most detailed collection of branch-level spectra to date and includes both common and biodiversity-indicator species. By making these measurements publicly available, the dataset supports the development of physically-based forest reflectance models, enables more accurate assessments of canopy structure and composition in boreal ecosystems, and improves forest and ecosystem monitoring through remote sensing.

## 3 Data Description

The dataset is available on Mendeley Data repository [3]. It includes:

1. “contact_probe_spectral_measurements.csv”: A table containing conical-conical reflectance factor (CCRF, [4]) spectra of tree branches, with one row per measurement. The first column provides the unique tree ID number, the second column specifies the tree species’ common name, the third column indicates the ID number of the sample’s position along the entire branch (e.g., 0–3), and the fourth column gives the ID number of the measurement’s position along the cut 30 cm long branch sample (e.g., 1–10). The remaining 2150 columns, labeled “wl[wavelength in nanometers]”, contain CCRF values measured at 1 nm intervals from 351 nm to 2500 nm. Fig. 1 illustrates the average CCRF spectra of the sampled branches calculated from the dataset.

**Fig. 1:**
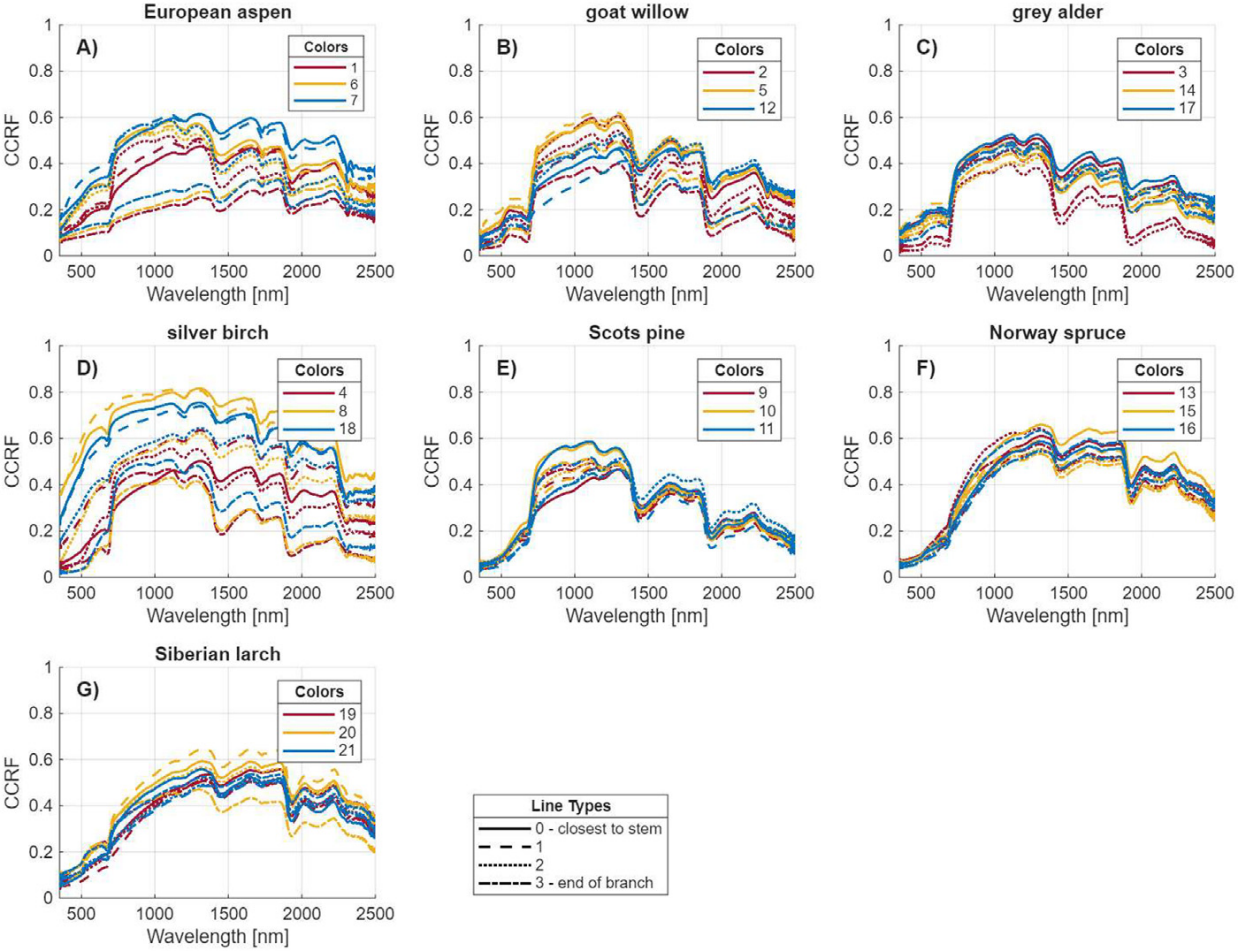
Average conical-conical reflectance factor (CCRF; 351–2500 nm) spectra measured with a contact probe for: a) European aspen, b) goat willow, c) grey alder, d) silver birch, e) Scots pine, f) Norway spruce, and g) Siberian larch. The four different line types labeled “0” to “3” denote the four sampling positions along each branch, collected from three trees per species. The three different trees are visualized in different colors that correspond to a unique tree ID in the dataset. The samples “0” (diameter > 2.5 cm) were taken nearest to the stem, while samples “3” (diameter > 0.5 cm) were from the distal end. Samples “1” and “2” were taken from evenly spaced positions between “0” and “3”.
2. “integrating_sphere_spectral_measurements.csv”: A table containing directional-hemispherical reflectance factor (DHRF, [4]) spectra, with one sample per row. The first column provides the unique tree ID number, the second column specifies the tree species’ common name, the third column indicates the ID number of the sample’s position along the entire branch (e.g., 0–3), and the fourth column gives the ID number of the measurement’s position along the cut 30 cm long branch sample (e.g., 1–10). Note that the integrating sphere measurements were taken only at the position closest to tree stem (position “0”), because the sample port size (6 mm in diameter) of the integrating sphere did not allow to reliably measure smaller branches. The remaining 2150 columns (named “wl[wavelength in nanometers]”) correspond to DHRF values measured at spectral bands from 351 nm to 2500 nm. Fig. 2 illustrates the average DHRF spectra of the sampled branches calculated from the dataset.

**Fig. 2:**
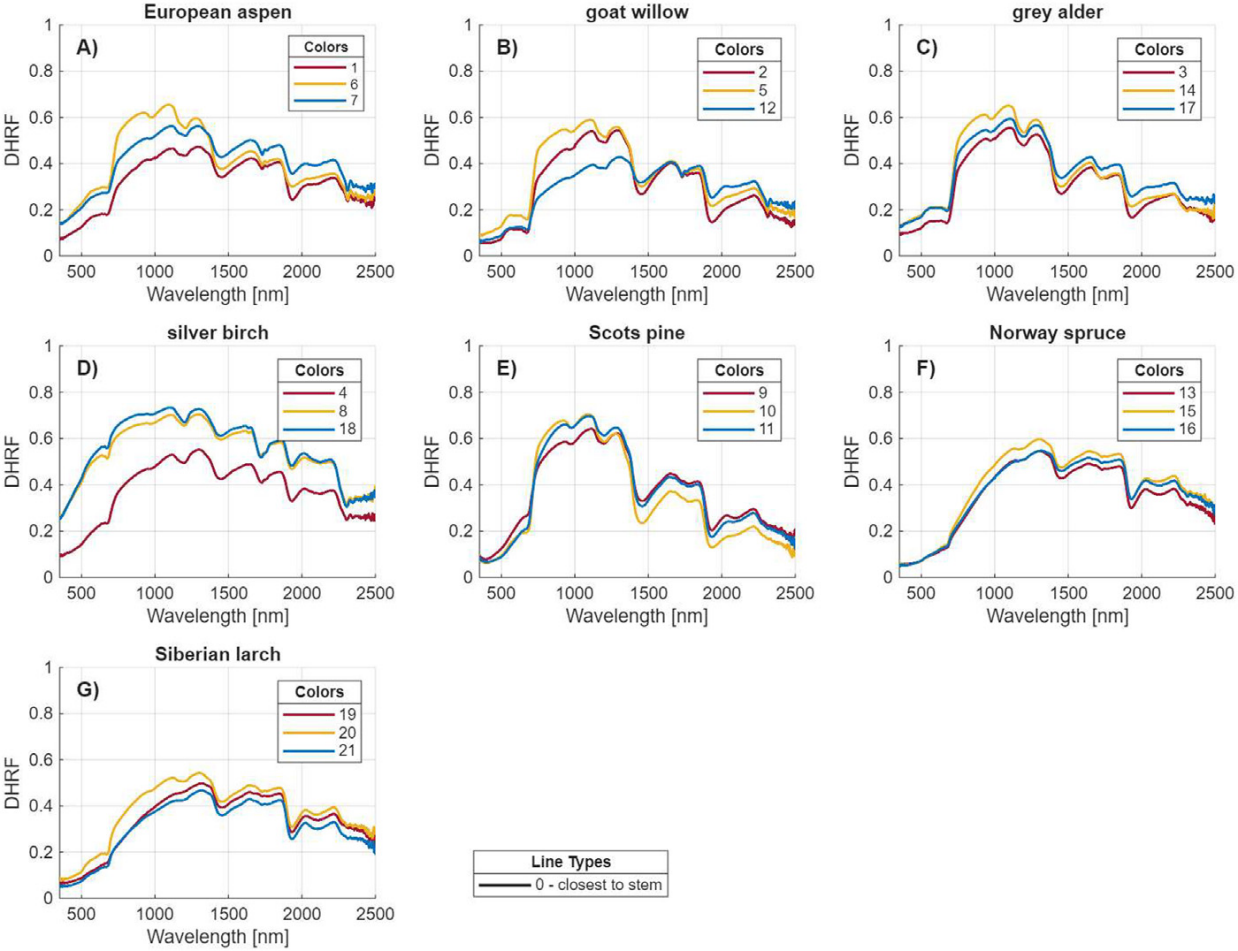
Average directional-hemispherical reflectance factor (DHRF; 351–2500 nm) spectra measured for samples collected from the base of the branch (“0”, diameter > 2.5 cm, closest to the stem) using an integrating sphere for a) European aspen, b) goat willow, c) grey alder, d) silver birch, e) Scots pine, f) Norway spruce, and g) Siberian larch. The three different trees per species are visualized in different colors and the corresponding unique tree ID are also shown.
3. “sampled_tree_descriptions.csv”: Table containing auxiliary data per each sample tree (see Table 1 for details).

**Table 1:** Column names and corresponding metadata for the “sampled_tree_descriptions.csv” file included in the dataset.

Column name	Description
tree_ID	Tree unique identifier
tree_species	Common name for the species of the sample tree
site	Location identifier
date	Date of sampling [YYYYMMDD]
dbh	Diameter at breast height (1.3 m) of sample tree [cm]
tree_height	Total height of sample tree [m]
branch_height	Height of cut branch from ground [m]
crown_base_height	Height of living crown base from ground [m]
branch_total_length	Total length of cut branch [m]
branch_length_to_0_5cm	Branch length from base to 0.5 cm diameter point [m]
4. “sample_photos.pdf”: Document showing photographs of the measured branch samples and the trees they were detached from (Fig. 3).

**Fig. 3:**
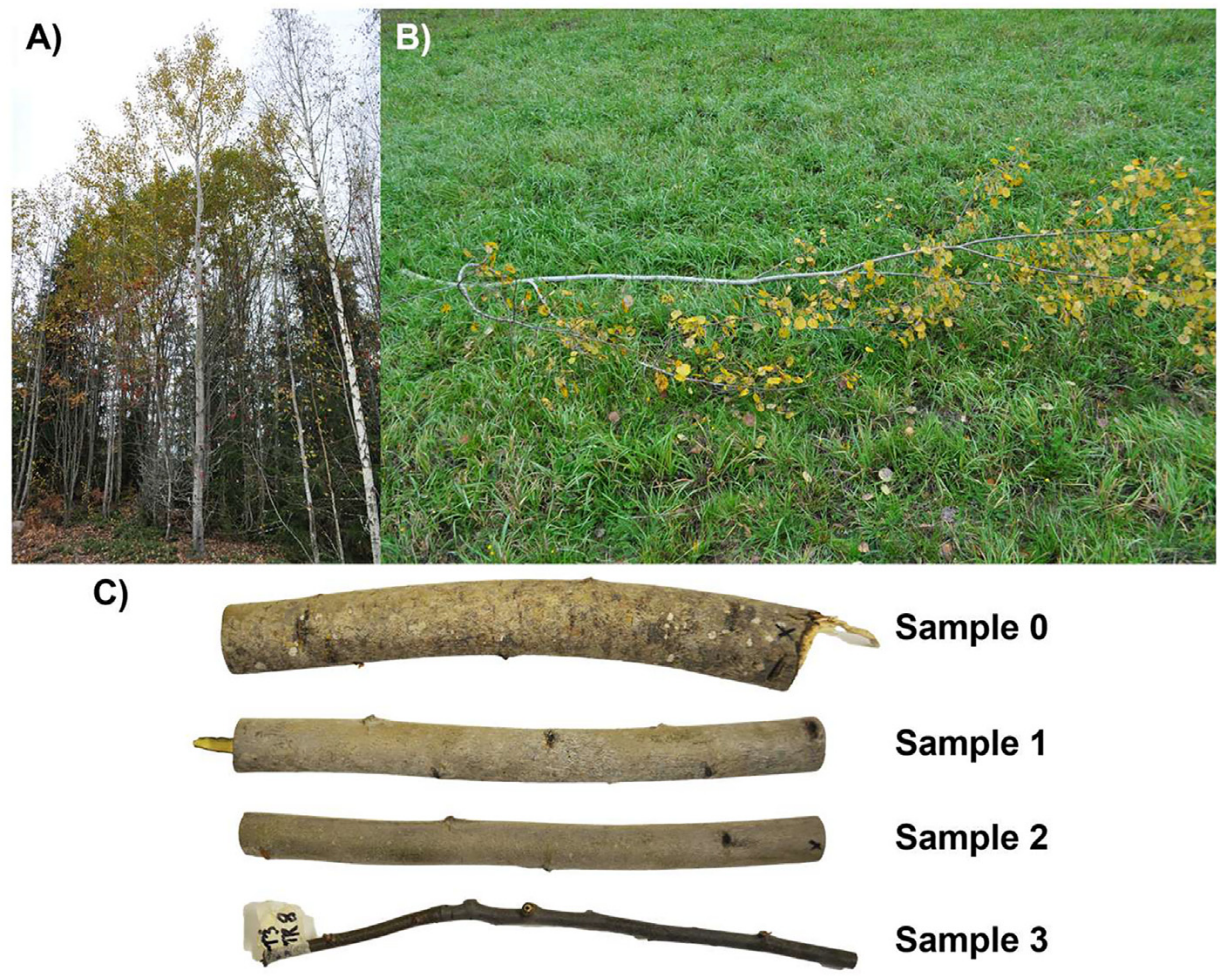
Example photographs of a European aspen (*Populus tremula* L.) showing a) the sample tree, b) the entire cut branch, and c) the samples taken from different positions along the branch: “0” (closest to the stem), “1”, “2”, and “3” (tip of the branch).

## 4 Experimental Design, Materials and Methods

### 4.1 Sampling

**Fig. 4:**
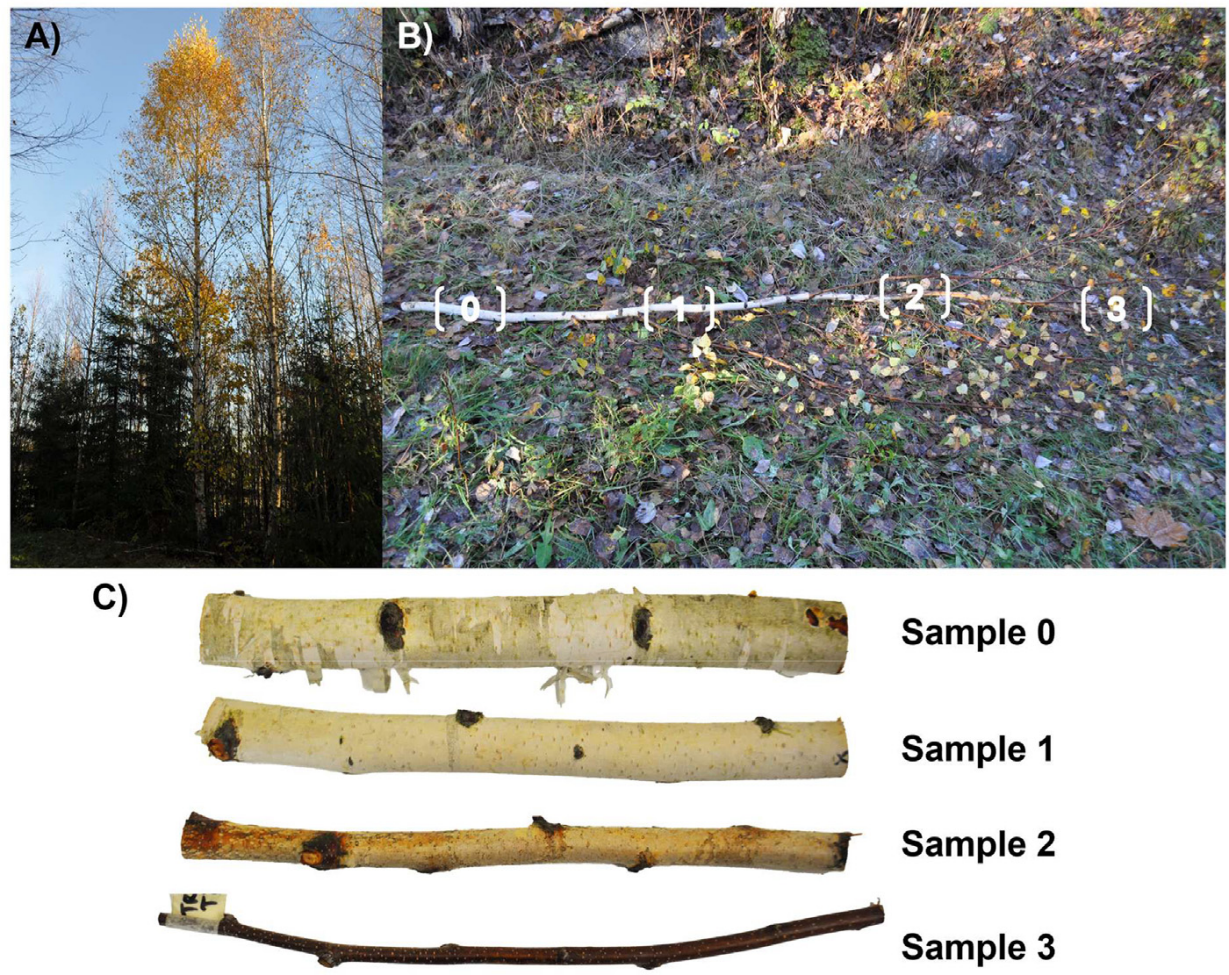
Visual examples of A) a sample tree, B) a branch cut from the middle of the living crown, near the 50 % relative height between the crown base and the treetop, and C) the four samples cut from each branch: one near the stem (“0”, diameter > 2.5 cm), one from the first tertile (“1”), one from the second tertile (“2”), and one near the tip (“3”, diameter > 0.5 cm).

Laboratory measurements of branch reflectance were conducted between 24 September to 19 November 2024 for samples from seven boreal and temperate tree species: European aspen*,* goat willow*,* grey alder*,* silver birch*,* Scots pine*,* Norway spruce*,* and Siberian larch. The Siberian larch samples were collected from the Viikki arboretum in Helsinki, Finland (60° 13′N, 25° 00′E), while samples from the other six species were collected from managed forests in Lohja, Finland (60° 23′N, 23°53′E). For each species, branches were sampled from three trees (21 trees total, tree height ranges per species: European aspen 14.5–16.9 m, goat willow 10.2–13.0 m, grey alder 9.2–18.7 m, silver birch 14.6–23.6 m, Scots pine 14.9–16.0 m, Norway spruce 21.9–26.5 m, Siberian larch 28.0–29.1 m) (Fig. 4A). One branch was cut per tree from the middle of the living crown, near the 50 % relative height between the crown base and the treetop, ensuring the branch was accessible and at least 2.5 cm in diameter (Fig. 4B). The lowest 2 m of the tree crown was avoided, as the branches there are usually thin and/or dead. From each selected branch, four 30 cm long samples were cut from the branch: one near the stem (“0”, diameter > 2.5 cm), one from the first tertile (“1”), one from the second tertile (“2”), and one near the tip (“3”, diameter > 0.5 cm) (Fig. 4C). The samples cut in the field were transported to a laboratory facility at Aalto University and cold-stored in a refrigerator prior to spectral measurements. Only dry-surfaced samples were measured, and surface areas with visible external material (e.g., lichens, mosses, and needles) were avoided. Two different spectral measurements were performed on the upper side of the branch: 1) all “0–3” samples were measured using a contact probe setup and 2) “0” samples were additionally measured with an integrating sphere setup to allow comparison of data acquired with different view geometries (conical vs. hemispherical). Data acquired in hemispherical view geometry are typically used as input in radiative transfer models, however, there does not exist integrating spheres that would have sample port small enough for measuring the smallest branches. The two setups are described more in detail below.

### 4.2 Spectral laboratory measurements and data processing

**Fig. 5:**
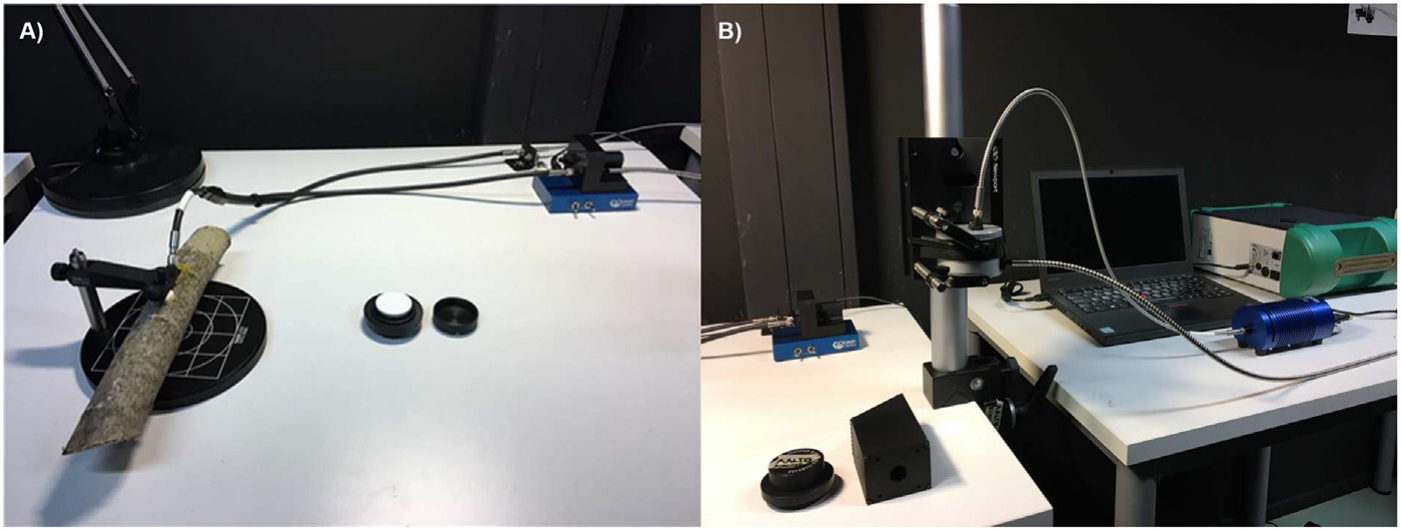
Photographs of the laboratory setups used for measuring branch reflectance. A) Contact probe setup with a 45° view angle using a Thorlabs Bifurcated 19-Fiber Bundle connected to an ASD FieldSpec 4 spectroradiometer and an Ocean Optics HL-2000 halogen light source. B) Integrating sphere setup using an Avantes Avasphere-30 connected to the same spectroradiometer and light source.

**Fig. 6:**
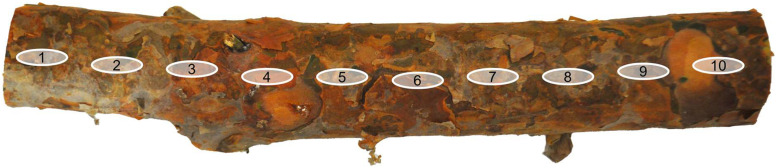
Visual illustration of the ten spectral measurements taken evenly along the upper surface of each 30 cm long branch sample. Measurement locations were occasionally adjusted to avoid surface areas with visible external materials, such as lichens, mosses, or needles.

For the contact probe spectral measurements, conical-conical reflectance factors (CCRF) were measured in a darkroom using a Thorlabs Bifurcated 19-Fiber Bundle (FG200LEA-FBUNDLE) connected to an Analytical Spectral Devices (ASD) FieldSpec 4 Standard-Res spectroradiometer (serial no 18,456) and an Ocean Optics HL-2000 tungsten halogen light source (Fig. 5A). The fiber bundle was mounted on a Thorlabs reflection probe stand (RPS-SMA) and positioned at a 45-degree angle relative to the sample surface. The receiver end of the fiber bundle had a conical field-of-view defined by a numerical aperture of 0.22, corresponding to a field-of-view of 25.42 degrees. The measurement distance was 4.8 mm, resulting in an elliptical illumination footprint of approximately 2.2 mm wide on the branch surface. The CCRF were recorded at wavelengths from 351 nm to 2500 nm. The spectral resolution of the instrument was 3 nm in visible (VIS) to near-infrared (NIR) wavelengths (≤ 1000 nm) and 10 nm in the shortwave-infrared (SWIR) wavelengths (> 1000 nm). The spectroradiometer interpolated and output the data at 1 nm intervals. Each spectral measurement sequence included three white reference, ten sample, and five dark current measurements. The ten measurements were distributed as evenly as possible along the upper surface of each branch sample (Fig. 6).

CCRF was then calculated using the following equation:

(1) $$\text{CCRF}\left(\lambda \right)=\;\frac{DN_{sample}\left(\lambda \right)-DN_{dc}\left(\lambda \right)}{DN_{wr}\left(\lambda \right)-DN_{dc}\left(\lambda \right)}\times R_{wr}\left(\lambda \right),$$

where DN*_sample_* and DN*_wr_* are the wavelength (λ) dependent digital number (DN) readings from the sample and average white reference measurements, respectively, and DN*_dc_* is the average DN readings of the dark current measurements. *R_ref_* is the calibrated reflectance factor of the Spectralon white reference panel (Labsphere Inc., serial no.: 99AA02–1115–3079 and 99AA03–0416–4533), included as a correction term to account for the non-ideal reflectance properties of the panel (nominal reflectance of 99 %).

For the integrating sphere measurements, directional-hemispherical reflectance factors (DHRF) were measured using an integrating sphere (Avantes Avasphere-30) connected to the same spectroradiometer and light source as used for the contact probe measurements (Fig. 5B). The diameter of the integrating sphere’s measurement port was 6 mm. In addition to the same white reference and sample measurements as with the contact probe setup, the stray light of the integrating sphere was measured using a Thorlabs Beam Trap (BT610/M). DHRF was calculated using the equation:

(2) $$\text{DHRF}\left(\lambda \right)=\;\frac{DN_{sample}\left(\lambda \right)-DN_{stray}\left(\lambda \right)}{DN_{wr}\left(\lambda \right)-DN_{stray}\left(\lambda \right)}\times R_{wr}\left(\lambda \right),$$

where DN_stray_ is the average of five DN readings from the stray light measurement. The spectral DHRF values in Eq. (2) were further corrected for the substitution error of the integrating sphere, using the following equation:

(3) $$\text{DHR}F_{corr}\left(\lambda \right)=\frac{\text{DHRF}\left(\lambda \right)}{1+k\left(\lambda \right)\left(\text{DHRF}\left(\lambda \right)-R_{wr}\left(\lambda \right)\right)}\;$$

The wavelength-dependent correction factor *k* was derived from measurements of calibrated Spectralon grayscale reflectance standards with nominal reflectance values of 5 %, 10 %, 20 %, 50 %, and 75 %.

All data processing were carried out using MATLAB (Version: 25.1.0.2973910 (R2025a) Update 1).

### 4.3 Auxiliary field measurements

Auxiliary measurements in the field included diameter at breast height (1.3 m), measured with a Haglöf Sweden tree caliper; total tree height, branch height, and crown base height, measured using a Haglöf Sweden Vertex 5; and total branch length and length of branch measured from the point of attachment to where diameter was 0.5 cm, measured with a tape measure. Note that the point where the branch diameter was 0.5 cm was the thinnest point where the smallest samples (i.e., sample 3) were cut from (Fig. 4).

## Limitations

None

## Ethics Statement

The current work does not involve human subjects, animal experiments, or any data collected from social media platforms.

## Credit Author Statement

**JJ:** conceptualization, investigation, methodology, software, data curation, writing – original draft; **MR:** conceptualization, investigation, methodology, resources, supervision, funding acquisition, writing – review & editing; **AH:** conceptualization, investigation, methodology, supervision, project administration, funding acquisition, writing – review & editing.

## Data Availability

Mendeley DataA dataset of branch reflectance spectra for seven boreal and temperate tree species (Original data).
